# AptoDetect™-Lung Assay as a Blood-Based Predictor of Advanced-Stage Lung Cancer in Patients with Lung-RADS 3–4 Pulmonary Nodules: A Multicenter Prospective Cohort Study

**DOI:** 10.3390/biomedicines14051013

**Published:** 2026-04-29

**Authors:** Bora Lee, Chi Young Kim, Jung Seop Eom, Wonjun Ji, Min Jee Kim, Sung Hoon Yoon, June Hong Ahn, Jun Hyeok Lim, Chaeuk Chung, Dong Won Park, Seung Hyeun Lee, Chang Dong Yeo

**Affiliations:** 1 Division of Pulmonary, Critical Care and Sleep Medicine, Department of Internal Medicine, Eunpyeong St. Mary’s Hospital, College of Medicine, The Catholic University of Korea, Seoul 03312, Republic of Korea; br9347@naver.com; 2 Division of Pulmonology, Department of Internal Medicine, Gangnam Severance Hospital, Yonsei University College of Medicine, Seoul 06273, Republic of Korea; cykim@yuhs.ac; 3 Department of Internal Medicine, Pusan National University School of Medicine, Busan 49241, Republic of Korea; ejspulm@gmail.com; 4 Division of Pulmonology and Allergy, Department of Internal Medicine, Asan Medical Center, University of Ulsan College of Medicine, Seoul 05505, Republic of Korea; wonjunji@amc.seoul.kr (W.J.); minjee60864@gmail.com (M.J.K.); 5 Division of Pulmonology, Department of Internal Medicine, Pusan National University Yangsan Hospital, Pusan National University School of Medicine, Yangsan 50612, Republic of Korea; drysh79@gmail.com; 6 Division of Pulmonology and Allergy, Department of Internal Medicine, Yeungnam University Medical Center, Yeungnam University College of Medicine, Daegu 42415, Republic of Korea; fireajh@gmail.com; 7 Division of Pulmonology, Department of Internal Medicine, Inha University Hospital, Inha University School of Medicine, Incheon 22332, Republic of Korea; jhl@inha.ac.kr; 8 Division of Pulmonology and Critical Care Medicine, Department of Internal Medicine, College of Medicine, Chungnam National University, Daejeon 35015, Republic of Korea; universe7903@gmail.com; 9 Division of Pulmonology and Allergy, Department of Internal Medicine, Hanyang University Hospital, Hanyang University College of Medicine, Seoul 04763, Republic of Korea; dongwonpark@hanyang.ac.kr; 10 Division of Pulmonary, Allergy and Critical Care Medicine, Department of Internal Medicine, College of Medicine, Kyung Hee University, Seoul 02447, Republic of Korea; 11 Department of Precision Medicine, Graduate School, Kyung Hee University, Seoul 02447, Republic of Korea

**Keywords:** lung cancer, pulmonary nodules, biomarker, aptamer, disease staging

## Abstract

**Background**: The AptoDetect™-Lung assay is an aptamer-based test designed for risk assessment in patients with pulmonary nodules, but its potential role in predicting lung cancer stage has not been evaluated. We investigated whether the assay could predict advanced-stage disease beyond conventional diagnostic modalities. **Methods**: This multicenter prospective cohort study enrolled 1672 patients with Lung-RADS 3–4 pulmonary nodules across ten university-affiliated hospitals in South Korea between June 2023 and December 2024. Among them, 934 patients with histologically confirmed lung cancer were retrospectively selected, and 852 patients were included in the final analysis after exclusions. The AptoDetect™-Lung assay was performed before invasive diagnostic procedures. **Results**: Among the 852 patients, 450 (52.8%) had advanced-stage disease. The AptoDetect™-Lung score was significantly higher in advanced-stage than in early-stage lung cancer (median, 6.2 vs. 2.8, *p* < 0.001). In a multivariable logistic regression analysis, a high AptoDetect™-Lung score (≥5) was independently associated with advanced disease (odds ratio 1.99, 95% confidence interval 1.35–2.95, *p* < 0.001). The AptoDetect™-Lung assay showed moderate discrimination of advanced-stage disease (area under the curve [AUC] 0.696) and in non–small cell lung cancer (AUC 0.720), whereas its discriminative ability was limited in small cell lung cancer (AUC 0.561). A combined prediction model incorporating the AptoDetect™-Lung assay, serum CEA, and radiologic findings demonstrated improved discriminative performance (AUC 0.821). **Conclusions**: The AptoDetect™-Lung assay score was independently associated with advanced-stage lung cancer and could provide clinically useful information for early risk stratification before definitive diagnosis and staging are available.

## 1. Introduction

Because low-dose chest computed tomography (LDCT) has demonstrated efficacy in lung cancer screening, reducing lung cancer mortality by 20%, it has been widely adopted in clinical practice [1]. However, LDCT has a high false-positive rate and often leads to additional unnecessary imaging studies or invasive diagnostic procedures, thereby increasing healthcare costs [2]. In addition, LDCT primarily detects pulmonary nodules and has limited ability to accurately assess nodal involvement or distant metastasis [3], which may result in underestimation of the true disease stage at the time of initial evaluation. Consequently, there has been growing interest in developing noninvasive adjunctive diagnostic tools such as blood biomarkers that could enhance diagnostic accuracy and reduce the clinical costs associated with lung cancer screening [4,5].

The AptoDetect™-Lung assay is a blood-based aptamer assay designed to assist in the diagnostic evaluation of patients with pulmonary nodules [6,7]. It detects seven protein biomarkers associated with lung cancer: EGFR1, MMP7, CA6, KIT, CRP, C9, and SERPINA3 [6]. Previous studies have reported that it provides favorable diagnostic performance in discriminating malignant from benign pulmonary nodules, especially within populations at high risk for lung cancer [6,8]. Given that it detects a combination of biomarkers released from lung cancer, it is reasonable to assume that it might be associated with disease burden and stage at diagnosis. In fact, current imaging-based staging of lung cancer may underestimate the true disease stage. For example, approximately 20% of patients who are initially considered to have clinical stage I are subsequently upstaged during further staging workup [9]. If a blood-based biomarker could aid in identifying patients with a high likelihood of advanced-stage lung cancer at the time of initial evaluation, it could facilitate more efficient diagnostic workup and potentially reduce delays in appropriate management.

The present study evaluated the association between the AptoDetect™-Lung score and lung cancer stage at diagnosis in patients with high-risk pulmonary nodules (classified as Lung Imaging Reporting and Data System [Lung-RADS] categories 3–4 [10]), aiming to assess its ability to discriminate early-stage from advanced-stage disease and to determine whether it provides additional information for staging.

## 2. Materials and Methods

### 2.1. Study Design and Cohort Selection

This multicenter prospective cohort study was conducted at ten university-affiliated hospitals in South Korea and prospectively enrolled 1672 patients between 13 June 2023, and 9 December 2024. Among them, 934 patients with histologically confirmed lung cancer were retrospectively selected for this analysis. Four patients without available staging data and 74 patients without AptoDetect™-Lung assay data were excluded, leaving 852 patients for the final analysis.

Demographic data, smoking history, comorbidities, occupational exposure, medication, laboratory data (AptoDetect™-Lung assay results and serum carcinoembryonic antigen [CEA] levels), and detailed chest CT findings were prospectively collected at enrollment. Fluorodeoxyglucose-positron emission tomography (FDG-PET) findings, histopathologic results, and final diagnosis with stage were obtained during follow-up.

Participants were eligible if they were aged 19 years or older and had pulmonary nodules categorized as Lung-RADS 3–4. Participants were excluded if they had pure ground-glass nodules, active pulmonary infection requiring treatment at enrollment, a history of histologically confirmed malignancy (with exceptions for malignancies in remission for >5 years, adequately treated non-melanoma skin cancer, localized prostate or thyroid cancer, or carcinoma in situ), or were pregnant. Pure ground-glass nodules were excluded because they often represent preinvasive or indolent lesions that may not release detectable circulating protein biomarkers. Patients with active pulmonary infection were excluded because inflammatory conditions may alter the levels of several proteins included in the assay. Patients with a history of malignancy were excluded to reduce the possibility that pulmonary nodules represented metastatic disease rather than primary lung cancer.

### 2.2. The AptoDetect™-Lung Assay and Sample Collection/Processing

The AptoDetect™-Lung assay is an aptamer-based liquid bead microarray test that quantifies seven target proteins (EGFR1, MMP7, CA6, KIT, CRP, C9, and SERPINA3) associated with lung cancer risk. The assay was performed using a bead-based multiplex platform with fluorescence detection on a Luminex system, and individual protein signals were integrated through a predefined algorithm to generate a composite risk score ranging from 0 to 10. An AptoDetect™-Lung score of 5 or higher was classified as high risk. Peripheral blood samples (4 mL) were collected at baseline before invasive diagnostic procedures and centrifuged to obtain serum, which was aliquoted and stored at −70 °C until analysis. To minimize variability between laboratories, all processed serum samples were sent to a central laboratory where the assays were performed. The assay can be completed within approximately 1.5 days once initiated, and the estimated cost is approximately USD 4.2 per test.

### 2.3. Definition of Early-Stage and Advanced-Stage Lung Cancer

Early stage was defined as stage I–II according to the eighth edition of the American Joint Committee on Cancer (AJCC) staging system for non-small cell lung cancer (NSCLC), and as limited disease for small cell lung cancer (SCLC). Pathologic staging was used for patients who underwent surgical resection, whereas clinical staging was used for those who did not.

### 2.4. Statistical Analysis

The Kolmogorov–Smirnov test was used to test data normality. Continuous variables were compared using Student’s *t*-test for normally distributed variables or the Mann–Whitney U test for non-normally distributed data. Categorical variables were compared using the chi-square test or Fisher’s exact test, as appropriate. Logistic regression analyses were performed to identify factors associated with advanced-stage lung cancer and construct a combined multimodal prediction model. Variables with a *p* value < 0.05 in univariable analyses were included in the multivariable model. Because several smoking-related variables showed significance in the univariable analyses and were highly correlated, only one representative smoking variable (smoking pack-years) was included in the multivariable model to minimize multicollinearity. Odds ratios (ORs) and 95% confidence intervals (CIs) are reported. Receiver operating characteristic (ROC) curve analyses were performed to evaluate the diagnostic performance of each modality in predicting advanced-stage lung cancer, and optimal cut-off values of lesion size, PET SUVmax, and serum CEA level for discriminating advanced-stage lung cancer were set using the Youden index. A two-tailed *p* value < 0.05 was considered statistically significant. All data analyses were performed using the R statistical package (version 4.5.2; R Foundation for Statistical Computing, Vienna, Austria).

## 3. Results

### 3.1. Patient Characteristics

Among a total of 852 patients with histologically confirmed lung cancer, 402 (47.2%) had early-stage disease and 450 (52.8%) had advanced-stage disease. By histology, 768 (90.1%) patients had NSCLC and 84 (9.9%) had SCLC. Baseline characteristics according to lung cancer stage are summarized in Table 1. Patients with advanced-stage lung cancer were more likely than those with early-stage disease to be male, have a lower body mass index, and be smokers. The AptoDetect™-Lung scores, serum CEA levels, maximum standardized uptake values (SUVmax) on FDG-PET, and Lung-RADS categories were all significantly higher in patients with advanced-stage lung cancer.

### 3.2. Association Between the AptoDetect™-Lung Assay and Lung Cancer Stage

**Figure 1:**
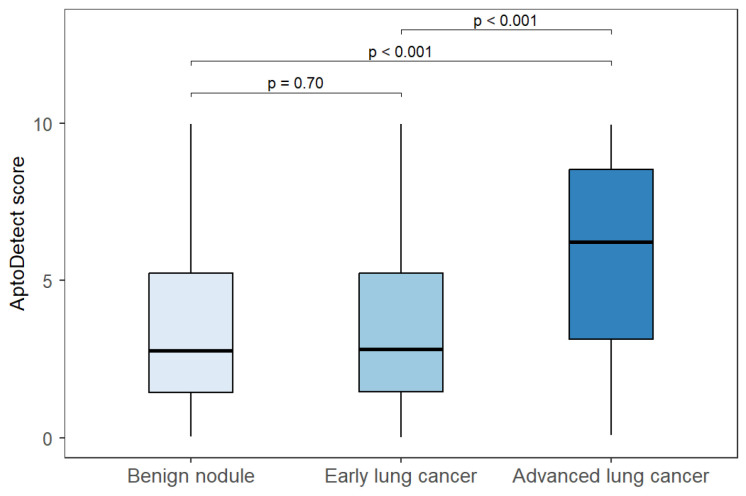
Comparison of the AptoDetectTM-Lung assay levels among benign nodules, early-stage lung cancer, and advanced-stage lung cancer.

The correlations between AptoDetect™-Lung assay scores and benign nodules, early-stage lung cancer, and advanced-stage lung cancer are compared in Figure 1. The AptoDetect™-Lung assay levels did not differ significantly between benign nodules and early-stage lung cancer, but the levels in advanced-stage lung cancer were significantly higher than those in the other groups (median 6.2 vs. 2.8, *p* < 0.001).

### 3.3. Predictors of Advanced-Stage Lung Cancer

**Figure 2:**
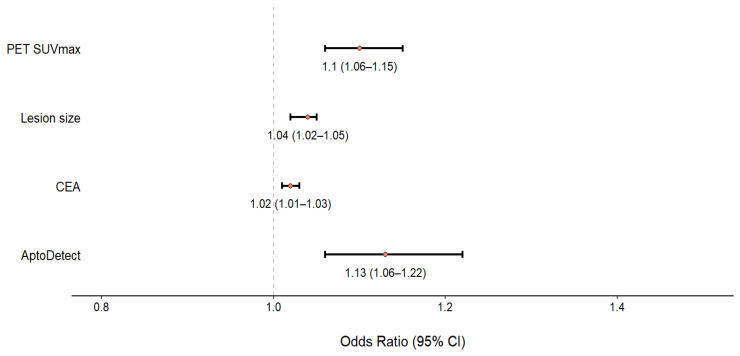
Key predictors of advanced-stage lung cancer. PET SUVmax, Maximum standardized uptake value on positron emission tomography; CEA, carcinoembryonic antigen; CI, confidence interval.

In the univariable logistic regression analyses, male sex, former or current smoking, SCLC, large lesion on CT, high AptoDetect™-Lung score, high PET SUVmax, and elevated CEA levels were significant predictors of advanced-stage disease (Table 2). In the multivariable logistic regression analysis, larger lesion size on CT, high AptoDetect™-Lung score, higher PET SUVmax, and elevated CEA levels remained independently significant predictors of advanced-stage lung cancer (Table 2, Figure 2).

**Figure 3:**
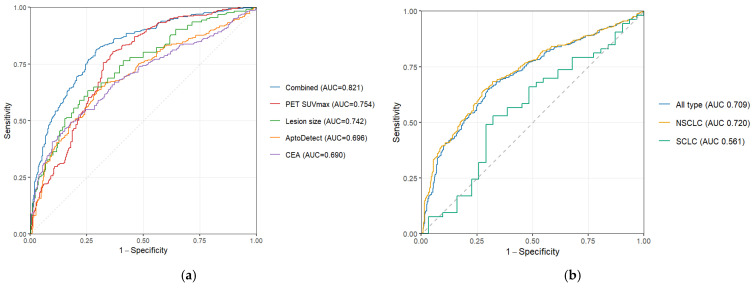
(**a**) ROC curves of multiple modalities for predicting advanced-stage lung cancer and (**b**) the AptoDetect^TM^-Lung assay for predicting advanced-stage lung cancer according to histologic subtype. The combined prediction model incorporated PET SUVmax, lesion size, the AptoDetect™-Lung assay, and serum CEA.

ROC curve analyses were performed to evaluate the diagnostic performance of the AptoDetect™-Lung assay and other modalities in predicting advanced-stage lung cancer (Figure 3a). The AptoDetect™-Lung assay demonstrated moderate discriminative ability, with an area under the curve (AUC) of 0.696. When stratified by histologic subtype, the diagnostic performance of the AptoDetect™-Lung assay was maintained in NSCLC (AUC 0.720) but was limited in SCLC (AUC 0.561) (Figure 3b). A combined multimodal prediction model incorporating the AptoDetect™-Lung assay score, CEA level, lesion size on CT, and PET SUVmax showed improved discriminative performance, compared with the individual modalities alone (AUC 0.821).

## 4. Discussion

In this study, we demonstrated an association between the AptoDetect™-Lung assay score and lung cancer stage and the additional value of the AptoDetect™-Lung assay in predicting advanced-stage lung cancer. Although the assay was originally developed to discriminate between benign nodules and lung cancer, our study suggests that its clinical utility could extend beyond malignancy detection to the assessment of disease extent at the time of diagnosis.

The AptoDetect™-Lung assay score showed an independent association with advanced-stage lung cancer after adjusting for sex, smoking history, cancer histologic type, and other staging-related diagnostic tests (OR 1.99 [95% CI 1.35–2.95]). In addition, it demonstrated discrimination of advanced-stage lung cancer (AUC 0.696) that was similar to that of the serum CEA level (AUC 0.690), which has modest diagnostic accuracy in lung cancer [11,12]. As a simple blood-based test that can be performed before definitive diagnosis, the AptoDetect™-Lung assay could provide clinically valuable information at a time when definitive staging information is not yet available, thereby enhancing pre-diagnostic risk stratification.

Furthermore, when it was incorporated into a combined prediction model with other diagnostic modalities, overall discriminative performance improved. This suggests that the assay could serve as a guiding tool in the early diagnostic phase and then continue to provide more accurate risk stratification as additional staging results are incorporated. A previous study of a multi-marker blood-based model that included CEA, CYFRA 21-1, and CA199 demonstrated modest predictive performance for intrapulmonary and distant metastasis (AUC 0.69), suggesting that circulating biomarkers can provide complementary information [13]. Other studies have shown that multimodal models that integrate both imaging-derived radiomics and clinical variables can improve staging-related prediction (AUC, 0.88–0.94), particularly for lymph node metastasis and discrimination between early-stage and advanced-stage disease [14,15,16]. However, those approaches typically require high-quality CT/PET-CT acquisition and standardized segmentation workflows, whereas our blood-based assay can be more readily deployable in the early diagnostic phase, complementing downstream imaging-based staging. Importantly, our results suggest that the AptoDetect™-Lung assay provides incremental value beyond conventional clinical factors, supporting its potential role as an adjunctive tool for early risk stratification.

Interestingly, the AptoDetect™-Lung levels did not differ significantly between benign nodules and early-stage lung cancer, whereas scores were significantly higher in advanced-stage disease. This stage-specific pattern can be interpreted in the context of blood-based biomarker biology: early-stage solid tumors might generate limited systemic molecular signals, making discrimination from benign conditions via circulating biomarkers challenging. Importantly, this finding does not necessarily indicate inadequate assay performance; rather, it suggests that the assay might reflect the systemic tumor burden or progression-related biology more than the mere presence of malignancy. If the AptoDetect™-Lung signal indeed captures disease extent, additional studies are warranted to determine whether it can also predict clinically relevant endpoints such as prognosis and treatment response, analogous to established serum tumor markers. In lung cancer, serum CEA has been associated with survival outcomes and has been investigated as a dynamic marker of treatment response in several studies, supporting the plausibility of extending biomarker utility beyond diagnosis to longitudinal monitoring [17,18,19,20,21].

Another notable finding is that the AptoDetect™-Lung assay showed relatively poor performance in predicting advanced stage in SCLC (AUC 0.561). One possible explanation is that the relatively small number of patients with SCLC was included in the analysis, which may have limited the statistical power and increased variability in performance estimates. In addition, approximately 70% of SCLC cases are diagnosed at an extensive stage, and even among patients with limited-stage disease, only about 5% present without nodal metastasis [22,23]. Consequently, the proportion of truly early-stage disease is very small, resulting in limited heterogeneity across disease stages. Therefore, compared with NSCLC, its ability to predict early-stage disease in SCLC appears to be inherently lower. 

Our study has several limitations. First, although the study cohort was prospectively enrolled across multiple centers, the analysis reported here was retrospective and therefore may be subject to selection bias and residual confounding. In addition, because the original cohort was designed to evaluate the diagnostic performance of the AptoDetect™-Lung assay for pulmonary nodules with suspected malignancy, the study population may include a relatively higher proportion of early-stage lung cancer compared with the broader lung cancer population. This may limit the generalizability of the findings across the full spectrum of lung cancer stages. Second, although the overall cohort size was relatively large, the number of patients in certain subgroups, particularly those with SCLC, was limited. In Korea, the proportion of SCLC among all lung cancers is relatively low, at approximately 13% [24,25]. This may reduce the statistical power for histology-specific analyses and could affect the precision and generalizability of estimates for these subgroups. Third, in most participants, the AptoDetect™-Lung assay was evaluated at a single time point at baseline; therefore, we could not assess whether longitudinal changes in the assay score reflect treatment response, disease progression, or prognosis. Finally, we did not evaluate the association between the assay and more granular stage-related outcomes, such as nodal stage (N stage) or detailed TNM subcategories. Therefore, the ability of the assay to discriminate specific patterns of locoregional or nodal involvement remains unclear.

## 5. Conclusions

In this study, the AptoDetect™-Lung assay score was independently associated with advanced-stage lung cancer, suggesting its potential role as a noninvasive adjunctive tool for identifying patients with a higher likelihood of advanced disease prior to definitive staging.

## Figures and Tables

**Table 1:** Baseline characteristics of the study population according to the stage of lung cancer.

Variables	Total (*n* = 852)	Early * (*n* = 402)	Advanced * (*n* = 450)	*p*-Value
Sex, male	575 (67.5%)	241 (60.0%)	334 (74.2%)	<0.001
Age	68.5 ± 9.8	68.6 ± 9.1	68.3 ± 10.5	0.63
BMI	23.8 ± 3.4	24.3 ± 3.5	23.4 ± 3.3	<0.001
ECOG				0.63
0–1	830 (97.4%)	390 (97.0%)	440 (97.8%)	
2–3	22 (2.6%)	12 (3.0%)	10 (2.2%)	
Smoking history				<0.001
Never	290 (34.0%)	166 (41.3%)	124 (27.6%)	
Current	312 (36.6%)	122 (30.3%)	190 (42.2%)	
Former	249 (29.2%)	114 (28.4%)	135 (30.0%)	
Smoking, pack-years	37.5 (22.5–49.0)	40.0 (20.2–46.0)	37.0 (22.5–50.0)	0.66
Cancer history	44 (5.2%)	18 (4.5%)	26 (5.8%)	0.15
Histology				0.06
NSCLC	768 (90.1%)	371 (92.3%)	397 (88.2%)	
ADC	522 (61.2%)	269 (66.9%)	253 (56.2%)	
SqCC	188 (22.1%)	82 (20.4%)	106 (23.6%)	
Others	58 (6.8%)	20 (5.0%)	38 (8.4%)	
SCLC	84 (9.9%)	31 (7.7%)	53 (11.8%)	
CEA	3.9 (2.2–9.5)	2.9 (1.9–4.9)	6.0 (2.9–24.1)	<0.001
PET SUVmax	8.7 (4.8–12.5)	5.5 (3.0–9.9)	11.0 (8.0–14.1)	<0.001
AptoDetect^TM^-Lung score	4.4 (2.0–7.5)	2.8 (1.5–5.2)	6.2 (3.1–8.5)	<0.001
Lesion size (mm)	35.8 ± 22.5	26.7 ± 14.2	43.9 ± 25.3	<0.001
Lung-RADS				<0.001
3	10 (1.2%)	10 (2.5%)	0 (0.0%)	
4A	61 (7.2%)	51 (12.7%)	10 (2.2%)	
4B	371 (43.5%)	211 (52.5%)	160 (35.6%)	
4X	410 (48.1%)	130 (32.3%)	280 (62.2%)	

* Early stage was defined as stage I–II for NSCLC and limited-stage disease for SCLC, whereas advanced stage was defined as stage III–IV for NSCLC and extensive-stage disease for SCLC. BMI, body mass index; ECOG, Eastern Cooperative Oncology Group; NSCLC, non-small cell lung cancer; ADC, adenocarcinoma; SqCC, squamous cell carcinoma; SCLC, small cell lung cancer; CEA, carcinoembryonic antigen; PET SUVmax, maximum standardized uptake value on positron emission tomography.

**Table 2:** Univariable and multivariable logistic regression analyses for predicting advanced-stage lung cancer.

Variable	OR (95% CI)	*p*-Value	Adjusted OR (95% CI)	*p*-Value
Sex (male)	1.92 (1.43–2.56)	<0.001	0.91 (0.54–1.64)	0.86
Age (≥65)	0.92 (0.69–1.23)	0.57		
BMI (≥18.5 kg/m^2^)	0.98 (0.54–1.79)	0.94		
Former smoker (ref. never)	2.08 (1.51–2.89)	<0.001		
Current smoker (ref. never)	1.59 (1.13–2.23)	0.008		
Smoking pack-years (≥20)	1.67 (1.27–2.19)	<0.001	0.90 (0.53–1.52)	0.69
Cancer history	1.33 (0.72–2.46)	0.369		
Cancer type (SCLC)	1.60 (1.00–2.54)	0.048	0.68 (0.36–1.27)	0.22
Lesion size (≥30 mm)	4.62 (3.46–6.17)	<0.001	2.43 (1.64–3.58)	<0.001
AptoDetect^TM^-Lung score (≥5)	3.97 (2.97–5.31)	<0.001	1.99 (1.35–2.95)	<0.001
PET SUVmax (≥7)	7.03 (4.92–10.03)	<0.001	3.96 (2.62–5.99)	<0.001
CEA (≥6 ng/mL)	4.41 (3.23–6.03)	<0.001	3.58 (2.38–5.38)	<0.001

OR, Odds ratio; CI, confidence interval; BMI, body mass index; SCLC, small cell lung cancer; PET SUVmax, maximum standardized uptake value on positron emission tomography; CEA, carcinoembryonic antigen.

## Data Availability

The data supporting the findings of this study are not publicly available due to patient privacy concerns and institutional restrictions. De-identified data may be made available from the corresponding author upon reasonable request and with appropriate institutional approvals.

## Abbreviations

The following abbreviations are used in this manuscript:

AUC	Area under the curve
LDCT	Low-dose chest computed tomography
Lung-RADS	Lung Imaging Reporting and Data System
CEA	Carcinoembryonic antigen
FDG-PET	Fluorodeoxyglucose-positron emission tomography
AJCC	American Joint Committee on Cancer
NSCLC	Non-small cell lung cancer
SCLC	Small cell lung cancer
OR	Odds ratios
CI	Confidence intervals
ROC	Receiver operating characteristic
SUVmax	Maximum standardized uptake value
